# Excitability of the Primary Motor Cortex Increases More Strongly with Slow- than with Normal-Speed Presentation of Actions

**DOI:** 10.1371/journal.pone.0114355

**Published:** 2014-12-05

**Authors:** Takefumi Moriuchi, Naoki Iso, Akira Sagari, Kakuya Ogahara, Eiji Kitajima, Koji Tanaka, Takayuki Tabira, Toshio Higashi

**Affiliations:** 1 Unit of Rehabilitation Sciences, Nagasaki University Graduate School of Biomedical Sciences, Nagasaki, Japan; 2 Medical Corporation Tojinkai Miharadai Hospital, Nagasaki, Japan; 3 Japanese Red Cross Society Nagasaki Genbaku Hospital, Nagasaki, Japan; 4 Faculty of Health and Social Work, School of Rehabilitation, Kanagawa University of Human Services, Kanagawa, Japan; 5 Center for Industry, University and Government Cooperation, Nagasaki University, Nagasaki, Japan; 6 Unit of Physical and Occupational Therapy, Nagasaki University Graduate School of Biomedical Sciences, Nagasaki, Japan; 7 Faculty of Rehabilitation Sciences, Nishikyushu University, Saga, Japan; University of Goettingen, Germany

## Abstract

**Introduction:**

The aim of the present study was to investigate how the speed of observed action affects the excitability of the primary motor cortex (M1), as assessed by the size of motor evoked potentials (MEPs) induced by transcranial magnetic stimulation (TMS).

**Methods:**

Eighteen healthy subjects watched a video clip of a person catching a ball, played at three different speeds (normal-, half-, and quarter-speed). MEPs were induced by TMS when the model's hand had opened to the widest extent just before catching the ball (“open”) and when the model had just caught the ball (“catch”). These two events were locked to specific frames of the video clip (“phases”), rather than occurring at specific absolute times, so that they could easily be compared across different speeds. MEPs were recorded from the thenar (TH) and abductor digiti minimi (ADM) muscles of the right hand.

**Results:**

The MEP amplitudes were higher when the subjects watched the video clip at low speed than when they watched the clip at normal speed. A repeated-measures ANOVA, with the factor VIDEO-SPEED, showed significant main effects. Bonferroni's post hoc test showed that the following MEP amplitude differences were significant: TH, normal vs. quarter; ADM, normal vs. half; and ADM, normal vs. quarter. Paired *t*-tests showed that the significant MEP amplitude differences between TMS phases under each speed condition were TH, “catch” higher than “open” at quarter speed; ADM, “catch” higher than “open” at half speed.

**Conclusions:**

These results indicate that the excitability of M1 was higher when the observed action was played at low speed. Our findings suggest that the action observation system became more active when the subjects observed the video clip at low speed, because the subjects could then recognize the elements of action and intention in others.

## Introduction

Recent years have seen great advances in brain imaging technology, enabling many researchers to elucidate brain mechanisms that had formerly been treated as black boxes. Rizzolatti et al. found neurons in area F5 of the monkey premotor cortex, now called “mirror neurons”, that fire both when the monkey performs specific goal-directed hand actions and when it observes another monkey or an experimenter performing the same or a similar action [1], [2]. Even with the same reaching and grasping action, it was revealed that mirror neurons in area F5 of the monkey premotor cortex are more activated when the monkey recognizes the underlying motor goals or when it understands the intentions associated with the actions of the model [3], [4]. Neurons having these characteristics have also been found in the inferior parietal lobule (area PF) and superior temporal sulcus (STS). STS is an important component of the perceptual system and the neurons within the STS have purely visual properties. [5]–[8]. These three areas were found to be mutually connected, and the entire neural circuit is called the “mirror neuron system” (MNS) [9], [10].

Moreover, a “mirror” system similar to that described in the monkey also exists in humans [11]–[13]. However, evidence for the existence of such a circuit is relatively weak [14]. Some studies have investigated neurons with both action-observation and action-execution properties in humans [15], [16]. However, limited evidence for these was found. Therefore, we refer to this neural circuit as the action observation system (AOS) in this paper. Evidence suggest that in the human brain, action observation recruits a consistent network of cortical areas, including the inferior frontal gyrus (within Broca's area; IFG) [17], [18], ventral premotor cortex (PMv) [19], inferior parietal lobule (IPL) [20], superior temporal sulcus (STS) (activated only in observation mode) [21], dorsal parietal lobule (PMd) [22], superior parietal lobule (SPL) [23], and anterior intraparietal area (AIP) [24].

The AOS is activated both when an action is performed and when the same action performed by others is observed, and these neurons are thought to be the basis of action recognition, action understanding, and automatic imitation [3], [4], [9], [13], [25]–[28]. Action observation reactivates the same cortical motor areas that are involved in the performance of the observed actions. Therefore, action observation shows promise as a potential tool for neurorehabilitation [29]–[33].

Consistent with the conclusions from AOS studies, transcranial magnetic stimulation (TMS) studies have shown that the excitability of primary motor cortex (M1) is enhanced during action observation [34]–[37]. This can be explained by the assumption that the PMv, which is an important node the AOS, and which has strong connections to M1, exerts an influence on M1 activity during the observation of actions [38]–[41]. Moreover, it has been demonstrated that M1 facilitation is modulated by the orientation of visual stimuli [39], the transitive/intransitive distinction in the observed movements [42], and the laterality of the observed body part [43].

However, many points concerning the details of modulation of the excitability of M1 during action observation require clarification. For example, there are doubts as to whether humans are, in fact, able to observe the details of quick and complex movements such as a figure skater's performance. In fact, judges in figure skating competitions often use slow-motion replay to help in making difficult evaluations [44]. In humans, the highest visual frequency that can be followed is 16 Hz, or a period of 0.06 s. Anything faster cannot be processed accurately [45]. For these reasons, human observational ability is thought to be limited, and it would therefore follow that we cannot register the details of quick and complex movements, casting doubt on the idea that the observation of such movements will help in re-acquiring them in a rehabilitation context.

Until now, the clinical application of action observation in the field of rehabilitation has consisted of showing the patient a video modeling the movement to be acquired. In our opinion, it is important that information obtained visually by patients demonstrate the elements of the movement to be acquired, without which it cannot be linked to motor learning. In fact, Parkinsonian patients respond to action observation only when the model is a patient with Parkinson's disease performing the action at a low, “bradykinetic” speed. Patients with PD do not respond to action observation when the action is performed at a higher speed by a neurologically healthy participant [46]. We predict that this effect is not specific to patients with PD, and that healthy participants will likewise respond preferentially to bradykinetic versions of actions. Therefore, observing the movements of others at low speed has possibilities for better assisting motor learning, on the assumption that patients are better able to recognize the elements of a movement. This leads to our hypothesis that the change in excitability of M1 during action observation will be different for different speeds of observed motion, for example, low speed versus normal speed.

The aim of this study was therefore to investigate the effect of the speed of the action on the enhancement of the excitability of M1 by action observation, as assessed by the amplitude of TMS-induced motor evoked potentials (MEPs). Any such effect would have great consequences for the development of novel neurorehabilitation strategies based on action observation.

### Subjects

Eighteen healthy volunteers (11 men and 7 women; mean age, 26.6 ± 6.8 years) were enrolled in this study after providing their written, informed consent. All subjects were self-reported right-handers. None of the subjects reported neurological impairment or contraindications to TMS [47], [48]. The study was approved by the local ethics committee at the Nagasaki University Graduate School of Biomedical and Health Sciences. All experimental procedures were conducted in accordance with the Declaration of Helsinki.

## Procedure

### 1) Experimental set-up

Subjects were seated on a reclining chair 80 cm away from a PC monitor (19-inch, resolution 1024 × 768 pixels, refresh frequency 60 Hz) and were instructed to keep both hands in a pronated position on a horizontal board attached to the chair's armrest. They were instructed to keep the right forearm as still and relaxed as possible while paying attention to the visual stimuli presented on the PC monitor. To ensure passive observation of the video clips, the experimenter's only instruction to the subjects was “You should stay alert while observing a hand,” before starting the experiment. Subjects were able to see their own hands. However, they were instructed to pay attention to the visual stimuli. Therefore, they sensed their hands in peripheral vision only.

### 2) Experimental stimuli

To create the stimulus video, a model was filmed from a first person viewpoint performing a one-handed ball-catching task using his right hand. An actor in the background threw the ball toward the model's location. The ball used in the video (S3C-NEW, Nagase-Kenko, Tokyo) is widely used in softball and is the official ball of the Japan Softball Association. The ball was #3 size (perimeter  =  30.48 ± 0.32 cm; weight, 90 ± 5 g; diameter, about 9.7 cm) and was colored black for purposes of distinguishing the ball from the color of the background. The video was recorded using a digital single-lens reflex camera (D5100, Nikon Corporation, Japan) and had a duration of 8040 ms (The total number of frames was 202.) The method of playing the video involved presenting a series of single frames, each lasting 40 ms, normal speed assumed (resolution 1,920 × 1,080 pixels, color depth 14 bits, frame rate 25 fps), fast enough to produce an animation effect.

### 3) Experimental procedure

**Figure 1:**
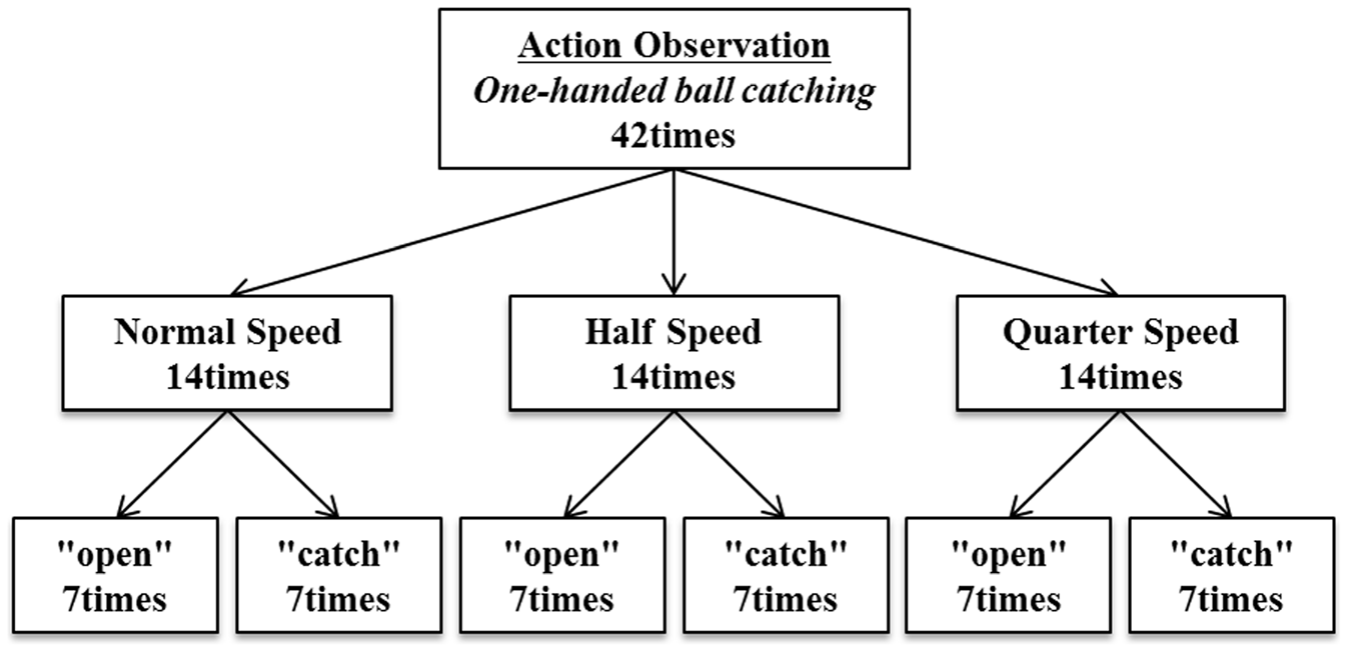
Experimental stimulation protocol. All subjects watched a video clip showing someone catching a ball one-handed, played at three different speeds (normal speed, half-normal speed, quarter-normal speed). In a given showing, TMS is delivered at one or another of two distinctive time points (“open” or “catch”). Speed conditions and the time point of TMS delivery were randomized across trials. TMS, transcranial magnetic stimulation.

Prior to the action observation task, the baseline excitability of M1 at rest was assessed in each subject by acquiring 14 MEPs under control conditions. Subsequently, the experimenter instructed the subject, “You should stay alert while observing a hand,” and all subjects then observed the same video clip played at three different speeds (normal-, half-, and quarter-speed conditions). Fig. 1 shows the experimental design. In this study, TMS was delivered to M1 in the left hemisphere during observation of the video and the MEP was evoked. Previous studies have found that MEP amplitude correlates significantly with finger aperture during grasping movements and peaks when finger aperture is maximal [49], [50]. For this reason, the present study adopted this time point, hereinafter designated as “open,” as a logical alternative to the moment of catch, here designated as “catch.”

The present study employed a 25 fps video clip taken with a digital camera. Individual frames were converted to jpeg files and shown in succession to obtain the animation effect. In the normal-speed condition, the presentation time of each frame was twice the length of the refresh interval used by the PC monitor (refresh interval  =  16.6 ms). Therefore, in the half-speed condition, the presentation time of each frame was four times the refresh interval, and in the quarter-speed condition, the presentation time of each frame was eight times the refresh interval.

The triggering of the TMS pulse was locked to the presentation of one or another of two specific frames in the movie, one frame showing the “open” time point and the other showing the “catch” time point. When one of these specially designated frames was output, the computer controlling the experiment recognized this and output a trigger pulse at the same instant. This design guaranteed that TMS relative timing would always be independent of presentation speed. (In addition, the word “phase” is more accurate than “time point” when comparing the same event across different speeds, and will be used in what follows.)

**Figure 2:**
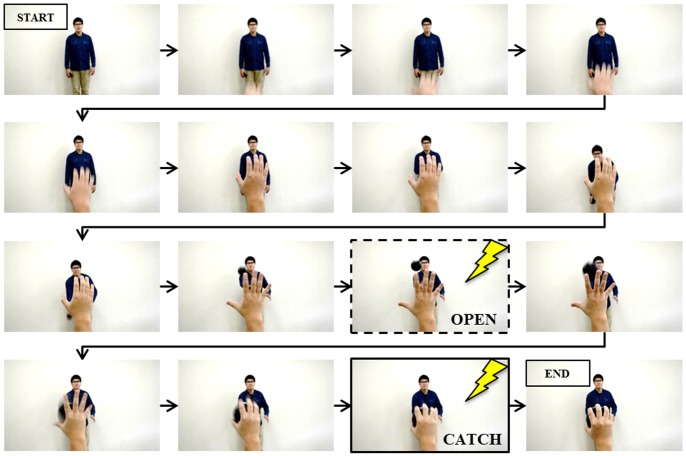
A sequence of stills from the video clip used in the experiment. The person in the background is the pitcher. The frame in the dashed box is at “open” and the frame in the solid box is at “catch.” TMS was triggered at one or another of these two time points in a given showing.

Fig. 2 shows 16 frames from the video clip used in this experiment. There were two timings of TMS delivery in the experiment. One was the frame inside the dashed box labeled “open,” and the other was the frame inside the solid box labeled “catch.” “Open” was at the time the model's hand had opened to the widest extent just before catching the ball (162/202 frames), and “catch” was at the time the model just caught the ball (172/202 frames). “The individual in this manuscript has given written informed consent (as outlined in PLOS consent form) to publish these case details.” TMS was delivered once for each video clip, randomly at “open” or “catch.” In each subject, each speed condition was used in 14 trials. Seven of these were trials that used the “open” phase of TMS delivery and seven used the “catch” phase. The MEP amplitudes were calculated for each TMS delivery. To ensure that TMS was always delivered at the correct time and that the experimental design was correctly implemented, we used a computerized pulse-generation system (Multi Trigger System, Medical Try System, Japan). The order of speeds (normal, half, or quarter) was randomized by the experimenter, and the order of timings of TMS delivery (“open” or “catch”) was randomized by the Multi Trigger System. Each of the six possible trial conditions (three levels of speed × two levels of phase) was replicated 7× in a subject, for a total of 14 trials per speed, and a further 14 trials were controls involving TMS and MEP data collection at intervals of 5 s during a sham trial, giving a total of 56 trials per subject.

### 4) TMS and MEP recording

Surface electromyogram (EMG) activity was recorded in the right thenar muscles (TH; the muscle serving thumb flexion, activating the “catch” phase) and in the right abductor digiti minimi muscle (ADM; the muscle serving little finger abduction, activating the “open” phase) using pairs of 9-mm-diameter Ag-AgCl surface cup electrodes (SDC112, GE Healthcare, Japan). The two test muscles chosen were maximally separated anatomically given that they were both intrinsic muscles of the hand, thereby minimizing crosstalk between recording sites.

Surface EMG signals were amplified with a bandwidth of 5–3000 Hz by using a digital signal processor (Neuropack sigma MEB-5504, Nihon Kohden, Japan), and were input to a computer for off-line analysis using an A/D converter (PowerLab16/30, AD Instruments, Australia).

At the beginning of the experiment, the optimal TMS coil position for evoking MEPs in both the right TH and the right ADM, called the “hotspot,” was found. TMS was delivered to the left M1 “hotspot,” marked with a pen on a swimming cap covering the scalps of the subjects. TMS employed a 70-mm figure-of-eight coil connected to a magnetic stimulator (Magstim200, Magstim, UK). The coil was placed tangentially to the scalp with its handle pointing backward and rotated approximately 45° away from the mid-sagittal line. Care was taken to maintain the same coil position relative to the scalp throughout the experiment. The resting motor threshold was defined as the lowest stimulus intensity that evoked an MEP at least 50 µV in amplitude in the right TH and ADM in five out of 10 trials. The test stimulus intensity was set at 110%–130% of the resting motor threshold and produced a >50-µV MEP with a success rate that ranged from 48% to 82% (mean, 62.0%). The mean size of the control MEP for the TH and ADM were approximately 0.5–1.0 mV. Throughout the experiments, the subjects were instructed to avoid inadvertent movements that could raise the EMG background. For each muscle in each trial, the 20 ms period preceding TMS triggering was checked for background EMG activity.

## Data Analysis

If background EMG was found, the data from both muscles in the trial were rejected. MEP amplitude (peak-to-peak) was measured over each muscle in every trial. MEP amplitude was expressed as percentage of the mean amplitude under control conditions.

The data were analyzed using a repeated-measures one-way ANOVA (video speed normal, half, and quarter) and paired *t*-tests. Bonferroni's post hoc test for multiple comparisons was used for further analyses. In all analyses, the *p* level for statistical significance was set at <0.05. All analyses were performed using statistical analysis software (SPSS version 19.0, IBM, USA).

## Results

### 1) Typical MEP waveforms

**Figure 3:**
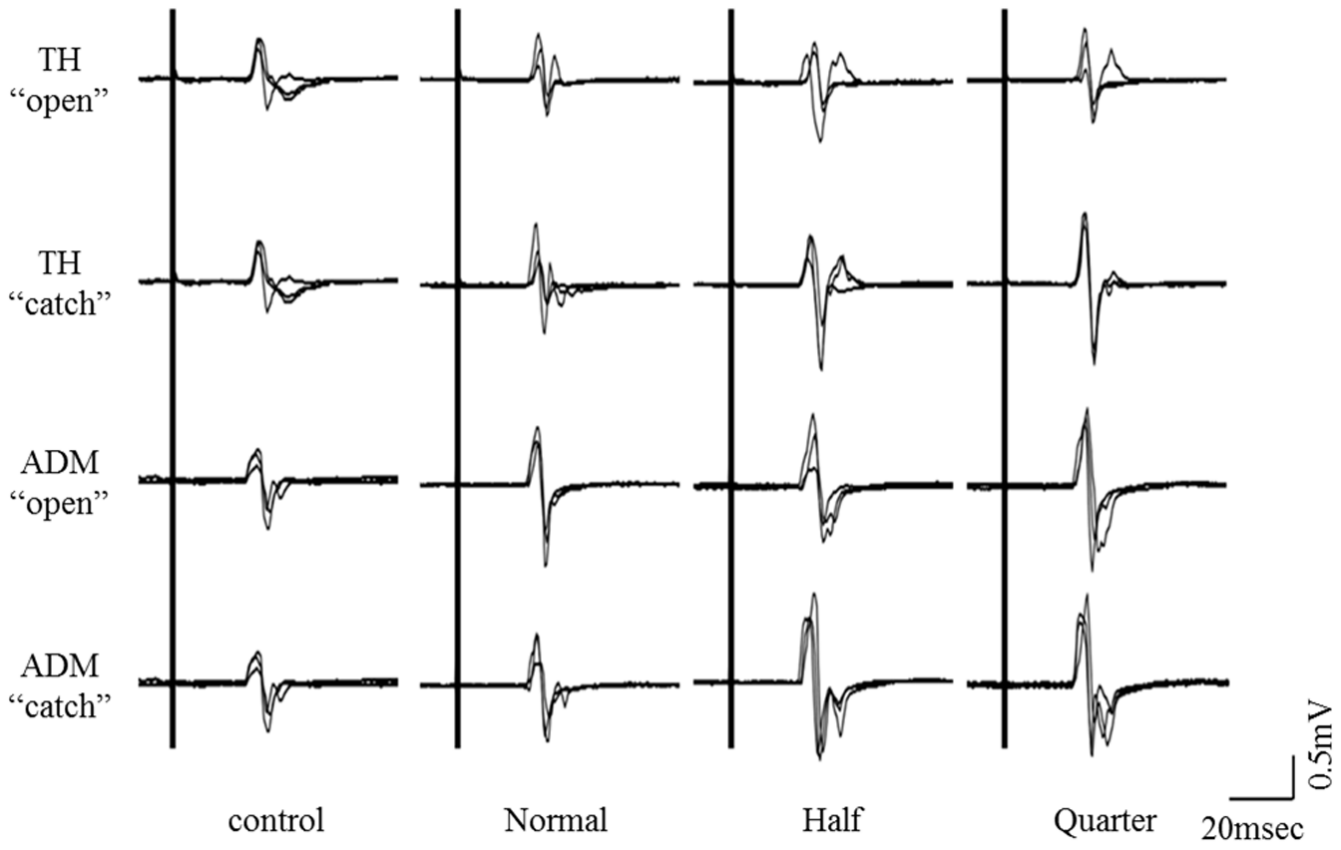
Typical MEP waveforms over the right TH and ADM as recorded from one representative subject. MEP amplitude is higher when the subject watches the video clip under low-speed conditions compared to watching the same clip played at normal speed. MEP, motor evoked potential; TH, thenar muscles; ADM, abductor digiti minimi muscle.

Typical MEP waveforms over the right TH and ADM, recorded from one representative subject, are shown in Fig. 3. There was a tendency for the MEP amplitudes in both muscles to be higher under task conditions than under control conditions. There was also a tendency for the MEP amplitudes in both muscles to be higher under low-speed conditions than under normal-speed conditions.

### 2) The mean MEP amplitude under each speed condition

**Figure 4:**
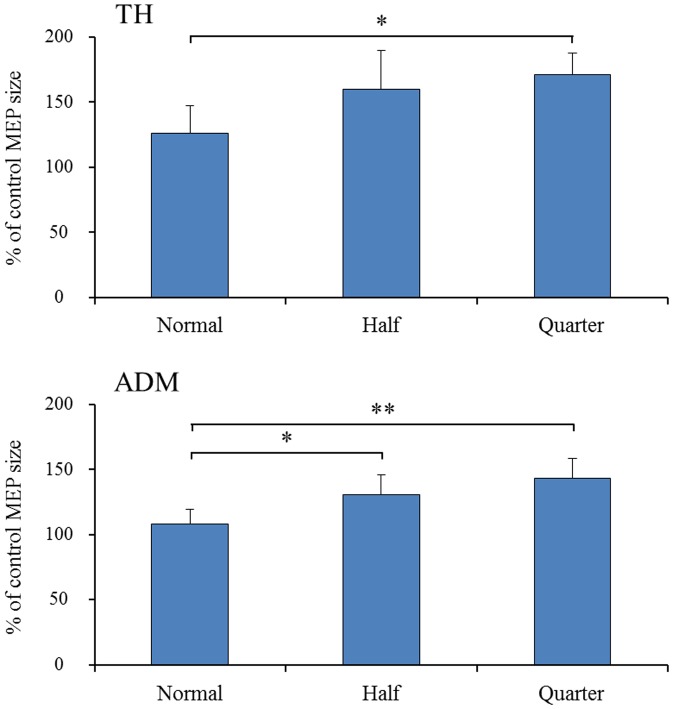
Mean MEP amplitude over the right TH and ADM under each speed condition. Values are expressed as percentage of the control-condition amplitude (n  =  18). To reveal the effect of speed, A one-way repeated measures analysis of variance was performed Asterisks indicate significant differences between speed conditions (*, *p* <0.05; **, *p* <0.01). Vertical bars indicate ± standard error.

The mean MEP amplitude as a percent of control (±SE) induced in the right TH and ADM in response to single-pulse TMS are shown for each muscle in Fig. 4. A one-way repeated measures analysis of variance was performed and a significant main effect of SPEED was found (three speeds: normal speed, one-half normal speed, and one-quarter normal speed). Significant main effects of SPEED were found in both muscles (TH, *F*  =  10.885, *p*  =  0.001; ADM, *F*  =  8.689, *p*  =  0.003). Among the three speeds, Bonferroni's post hoc test detected significant differences (shown in Fig. 4) in the TH between normal and quarter speed (*p*  =  0.025), in the ADM between normal and half (*p*  =  0.023), and in the ADM between normal and quarter speed (*p*  =  0.005).

### 3) The mean MEP amplitude under the difference of timing

**Figure 5:**
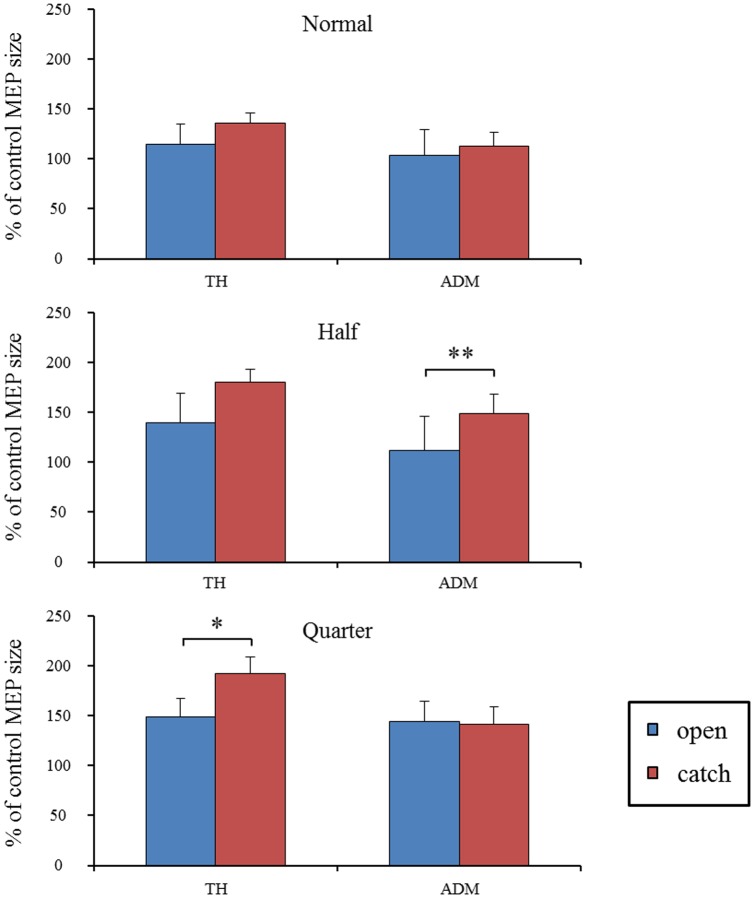
Mean MEP amplitude over the right TH and ADM under each speed condition in response to TMS. Values are expressed as percentage of the control-condition amplitude (n  =  18). To compare the difference in mean MEP amplitudes between the two TMS timings (“open” vs. “catch”) under each speed condition, the data were analyzed with paired *t*-tests. Asterisks indicate significant differences between speed conditions (*, *p* <0.05; **, *p* <0.01). Vertical bars indicate ± standard error.

The mean MEP amplitude as a percent of control (±SE) induced in the right TH and ADM in response to single-pulse TMS with different timings are shown in Fig. 5 for each speed condition. To compare the difference of mean MEP amplitudes between timings (“open” vs. “catch”) under each speed condition, the data were analyzed by paired *t*-tests. The MEP amplitude over the TH was significantly higher at the timing of “catch” than at the timing of “open” under the one-quarter normal speed condition (*p* <0.05). Moreover, the amplitude over the ADM was significantly higher at the timing of “catch” than at the timing of “open” in the half speed condition (*p* <0.01).

## Discussion

### 1) Differences in excitability of the primary motor cortex under different speed conditions

In the present study, we turned our attention to the issues raised for action-observation research by rapid movement. We investigated the effect of the speed of the action on the action-observation enhancement of M1 excitability, as assessed by the amplitude of TMS-induced motor evoked potentials (MEPs).

Some studies have shown that the excitability of M1 is enhanced by the social features characterizing an observed action. These studies suggest that the excitability of M1 is modulated by the participant's understanding of the social intention and context implied by the observed action [51]–[53]. In the present study, the experimental action video clearly presented someone catching a ball thrown by someone in front of him/her. Therefore, it was possible to understand the context and intention. For this reason, the excitability of M1 would have been enhanced by viewing the video according to these studies, which was indeed found.

MEP amplitude was found to be significantly different between the different speed conditions. The MEP amplitude was higher when subjects observed an action played at low speed relative to the normal-speed condition. For this reason, the present study revealed a positive effect of our manipulations of the viewed action speed on the excitability of M1 during passive observation of another's actions.

Some studies suggest that the changing excitability of M1 may be mediated by PMv mirror neurons modulating the activity of the motor cortical output [54]. Previous studies have found, using f-MRI, that the PMv, PMd, and IPL are particularly involved in the subject's ability to understand motor-related components of observed actions, and one author suggested that AOS activation is dynamically modulated by those aspects of actions that the subject is required to recognize [55]. For these reasons, we suggested that in action observation, the AOS would be activated by a reduction of the replay speed of a video clip of rapid movement because the subject would be able to register the elements of the action more easily.

Earlier TMS studies of action observation suggested that the parts of the viewer's M1 that control the particular muscles that the observed person activates are facilitated [12], [56]. The changes in M1 excitability were strongly muscle-specific; observation of an action that involves a particular muscle elicits a stronger response in the corresponding muscle of the observer than would observation of an action that does not use the muscle [38], [57]–[59]. Moreover, the change of M1 excitability was also phase-specific; it followed the kinematic profile of the observed movement [49], [50].

MEPs recorded over TH showed a tendency to greater amplitude at the timing of “catch” than at the timing of “open” under all speed conditions. Moreover, the amplitude difference reached significance under the quarter speed condition. From the TH results, we conclude that the excitability of M1 was modulated in a muscle-specific and phase-specific manner under the low speed conditions.

However, the ADM data showed that the excitability of the corresponding part of M1 was significantly higher at the timing of “catch” than at the timing of “open” in the half speed condition. However, there was no clear trend as in the TH data. Initially, we conducted the experiment under the assumption that ADM only participated in producing the “open” state. However, in practice, ADM also plays a role in metacarpophalangeal joint flexion in the little finger during “catch,” because of the resemblance of the latter state to a whole-hand grasp. For these reasons, we found the excitability of M1 to be similarly increased at both time points at the cortical representation of this muscle.

To summarize, our findings suggest that the excitability of M1 is facilitated by playing the video at a low speed, and we could recognize anatomical and phase elements of the movement at low speed that could not be recognized at normal speed, indicating that the AOS was strongly activated by slowing the presentation. However, because of deficiencies in the selection of test muscles and because of the use of only two TMS timings, the present study was incomplete and revealed only that the excitability of M1 was modulated in a muscle- and phase-specific manner. Therefore, a future reinvestigation with more attention to selection of the test muscles is needed. This study has only investigated speed reductions down to the one-quarter-normal speed condition. We were concerned that if the video playback speed were slowed down excessively, the subjects might have difficulty sustaining attention. Therefore, further studies are now needed to determine the optimal reproduction speed for activating the AOS during action observation.

### 2) Limitations

A previous study suggests that measurements of corticospinal excitability by TMS during action observation may be an excellent paradigm for probing the AOS [60]. However, the method used in that study could only probe the AOS indirectly and could not explore the functional roles of other cortical areas in which mirror neurons have been found, such as the PMv or AIP. Several current studies aim to explore the individual effects of various neural networks involved in the AOS during action observation directly, by means of the bifocal twin-coil TMS method. The bifocal twin-coil method is a conditioning-test TMS paradigm in which a test stimulus is applied over M1 at different delays after a conditioning stimulus is delivered over another cortical area [61]–[63]. The main result of a study that investigated the excitability of the connections linking AIP and PMv with M1, was that the excitabilities were not changed at rest, but they were facilitated in a muscle-specific way during action observation [63], [64]. In these reports, activation of the AOS during action observation has been shown to induce specific neurophysiological changes in some of the corticocortical connections of the human motor system, such as anterior intraparietal area-primary motor cortex (AIP-M1) and ventral premotor cortex-primary motor cortex (PMv-M1). This reinforces the idea that increased excitability of the motor cortex during action observation is mediated by activation of the AOS. There is a need for further study of the effect of the speed of the viewed action on the AOS, using the bifocal twin-coil TMS protocol, f-MRI protocol, or other methods.

In our study, we investigated M1 excitability during action observation only under normal versus slower-than-normal conditions. The reason we did not compare normal and faster-than-normal conditions was that the present study focused simply on activating the AOS during observation of what are seen as quick movements under natural conditions. The components of rapid motions such as a figure skating jump are more recognizable under slow-replay conditions. A previous study has suggested that the AOS is more modulated when the subject recognizes an aspect of the observed movement. Moreover, if we propose to apply action observation to a motor learning or rehabilitation paradigm, it makes no sense to incorporate artificially high-speed conditions. For these reasons, the present study did not investigate such high-speed conditions. However, if we want to reveal more about the relationship between the speed of movement and activation of the AOS, we will need to conduct a comprehensive examination with the addition of a faster-than-normal condition.

### 3) Summary and conclusions

Action observation reactivates the same cortical motor areas that are involved in the performance of the observed actions. Therefore, action observation has received considerable attention in the field of rehabilitation. Moreover, some studies have reported a therapeutic effect of action observation in randomized clinical trials in recent years [65], [66]. Although more study will be needed in the future, the present work reveals that activation of the AOS during observation of rapid movement is better under low-speed playback. For this reason, our findings lead us to recommend that, in the field of rehabilitation, if the patient is being asked to observe rapid movement, it should be shown in slow motion. Finally, we believe that the present study's findings could be relevant to extending the power of action-observation approaches to rehabilitation of stroke patients.
